# Salvage endoscopic nasopharyngectomy in locally recurrent carcinoma adjacent to the internal carotid artery—A case report

**DOI:** 10.1002/ccr3.9167

**Published:** 2024-07-07

**Authors:** Paraskevi Karamitsou, Alexandros Poutoglidis, Aikaterini Karamitsou, Georgia‐Evangelia Papargyriou, Argyro Leventi, Christos Georgalas

**Affiliations:** ^1^ Department of Otorhinolaryngology‐Head and Neck Surgery ‘G. Papanikolaou’ General Hospital Thessaloniki Greece; ^2^ Department of Anatomy and Surgical Anatomy, School of Medicine Aristotle University of Thessaloniki Thessaloniki Greece; ^3^ Vascular Centre Manchester Royal Infirmary Hospital, MFT NHS Trust Manchester UK; ^4^ Endoscopic Skull Base Centre Athens Hygeia Hospital Athens Greece; ^5^ Medical School University of Nicosia Nicosia Cyprus

**Keywords:** adenoid cystic carcinoma, case report, endoscopic nasopharyngectomy, internal carotid artery, nasopharyngeal malignancy

## Abstract

**Key Clinical Message:**

In cases adjacent to critical structures, such as the internal carotid artery, surgeons should meticulously explore the feasibility of surgery before declaring the neoplasm unresectable.

**Abstract:**

Salvage treatment for locally recurrent carcinoma of the nasopharynx constitutes a unique challenge. Surgery remains the gold standard treatment modality. Endoscopic nasopharyngectomy is considered a safe and feasible procedure overcoming the morbidities of an open surgery. Tumor adjacency to the internal carotid artery (ICA) is not an absolute contradiction for the endoscopic approach. Even in cases adjacent to critical structures, surgeons should meticulously explore the feasibility of surgery before declaring the neoplasm unresectable. We present the case of a 56‐year‐old male with locally recurrent adenoid cystic carcinoma (AdCC) of the nasopharynx adjacent to the ICA treated with endoscopic nasopharyngectomy.

## 1 INTRODUCTION

Adenoid cystic carcinoma (AdCC) is the most common malignant tumor of the minor salivary glands.^1^ Uncommon locations of the neoplasm in the head and neck area have been reported.^2^, ^3^ Although it is a slow‐growing tumor, it tends to recur locally and distally due to the high rate of perineural invasion. AdCC presents three main histological patterns: solid, tubular, and cribriform. Solid components are associated with the worst prognosis, but in most cases, the three patterns coexist.^4^

Salvage surgery for the resection of recurrent carcinoma of the nasopharynx may be performed either with the traditional open approaches or endoscopically. It can also be assisted robotically.^5^, ^6^, ^7^, ^8^ As open approaches are associated with cosmetic and functional morbidities, minimally invasive procedures have been employed in the management of recurrent carcinomas of the nasopharynx. Conservative treatment with radiotherapy or chemotherapy cannot be used in an already irradiated field, rendering surgery the only treatment option.^9^ Tumors of salivary gland origin demonstrate significant radioresistance, making radiotherapy incapable of a complete tumor response.

## 2 CASE HISTORY

A 56‐year‐old male presented to the ear, nose, and throat (ENT) outpatient clinic for his follow‐up appointment. The patient's medical history was remarkable for grade 1 AdCC of the nasopharynx. The AdCC was clinically staged as T3N0M0 (cT3N0M0) (Stage III). It had been treated with concurrent chemotherapy (14 cycles) and radiotherapy (33 sessions) 2 years ago. The patient was in a regular follow‐up for recurrence surveillance.

## 3 METHODS

**FIGURE 1:**
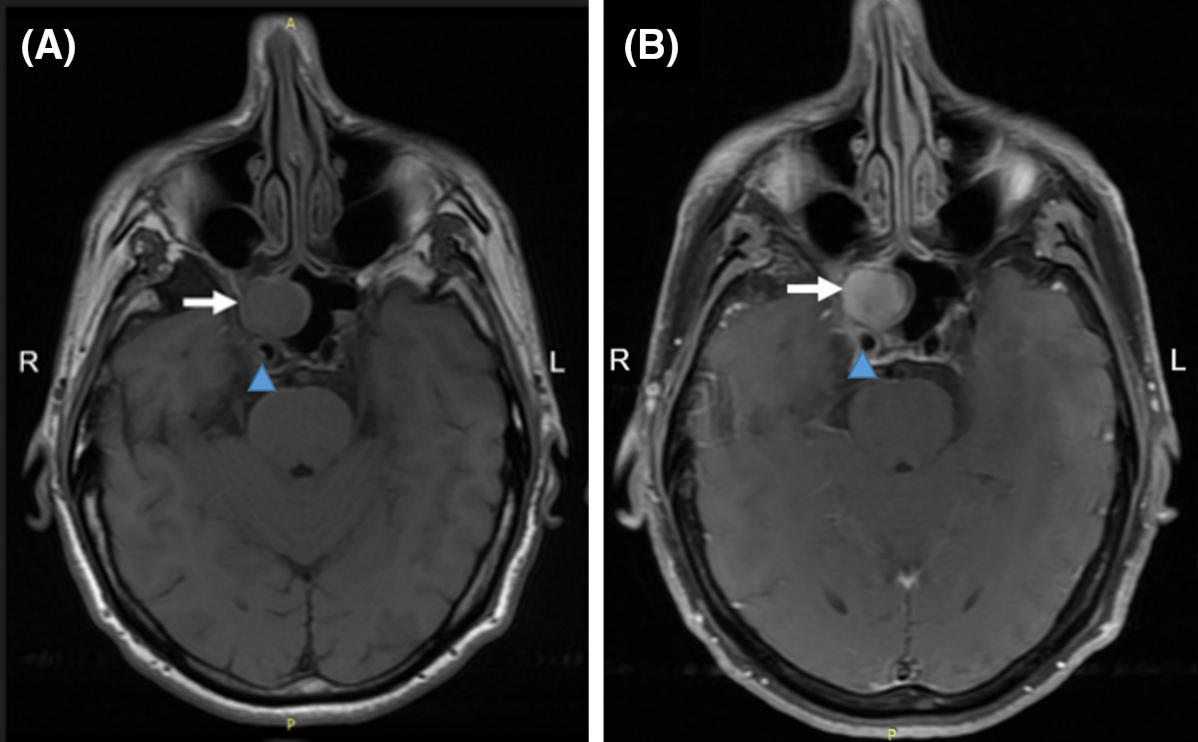
Sinus MRI. Tumor (white arrow) (2.0 × 1.8 × 1.6 cm) with invasion of surrounding structures. (A) Axial (T1W1 sequence), (B) Axial (T1W1 sequence with paramagnetic contrast agent administration). Blue arrowheads demonstrate the right ICA.

Nasal endoscopy revealed the presence of a mass in the right half of the posterior nasopharyngeal wall. Neck palpation did not reveal any abnormal findings. Further investigation with 18F‐fluorodeoxyglucose (18F‐FDG) positron emission tomography/computed tomography (PET/CT) showed increased metabolic activity with a maximum standard uptake value (SUVmax) of 3.9 in the aforementioned spot. The nasopharyngeal mass was biopsied under local anesthesia and the concomitant histology report indicated the presence of a recurrent AdCC with a combination of cribriform and tubular patterns. The proportion of cribriform patterns was less than 30%, grading the neoplasm as Grade 1. A magnetic resonance imaging (MRI) revealed tumor occupation of the right half of the posterior nasopharyngeal wall, expansion in the orifice of the right eustachian tube and the fossa of Rosenm*üller*, as well as partial infiltration of the clivus (Figure 1). Clinical staging was determined as recurrent T4bN0M0 (rT4bN0M0) (Stage IVb).

**FIGURE 2:**
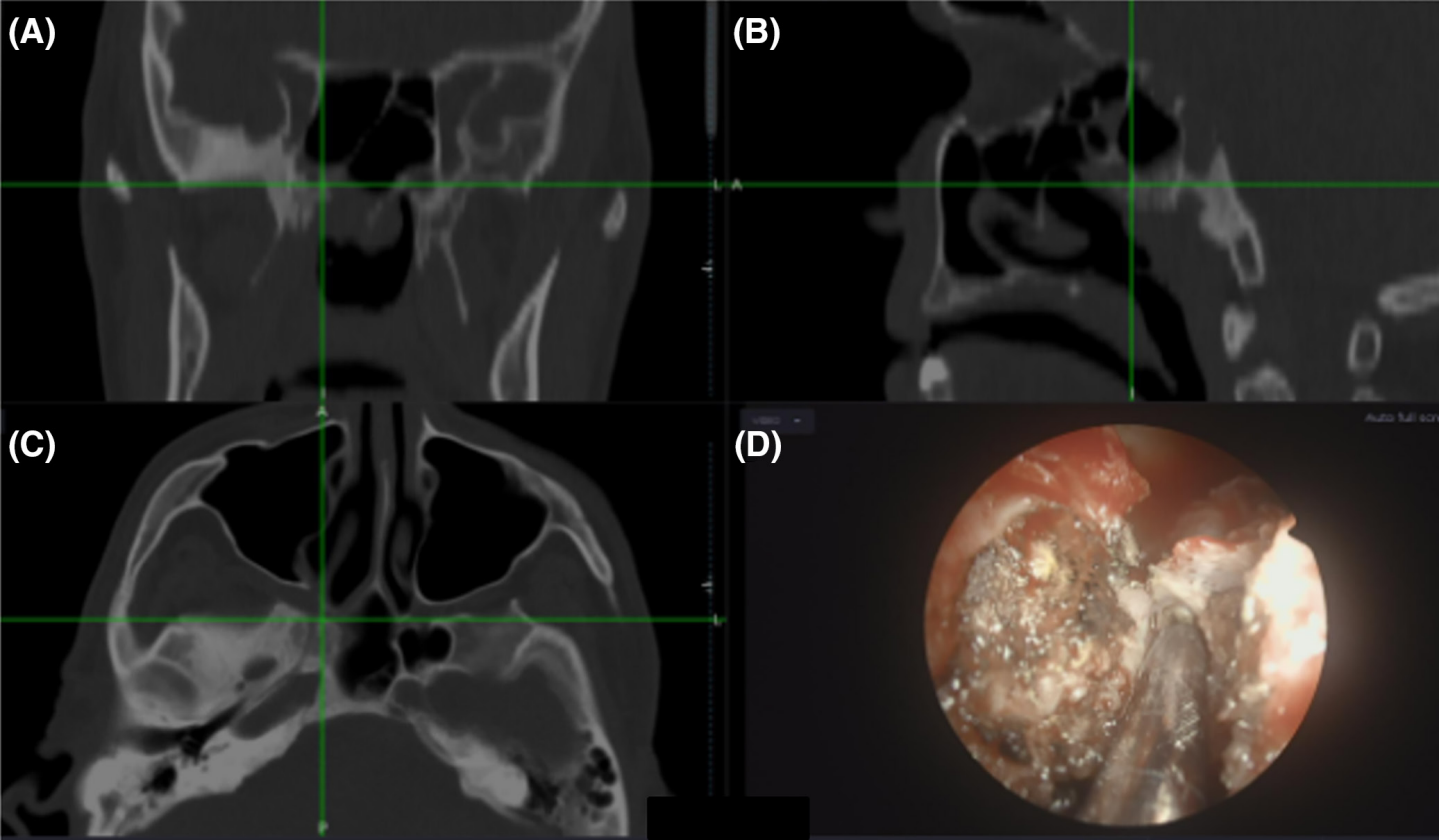
Image‐guided navigation system used reformatted thin‐cut CT scans in coronal (A), sagittal (B), and axial (C) sections to create a three‐dimensional portal. The neoplasm invaded the posterior wall of the right pterygopalatine fossa (A–C). A friable tumor is separated from the structures of the right pterygopalatine fossa as shown in the endoscopic image (D).

Our hospital's multidisciplinary team (MDT) assessed both the previous treatment performed and the feasibility of surgery and finally recommended surgical management. An endoscopic type III nasopharyngectomy was performed. The resection included middle maxillectomy and excision of the inner wall of the right maxillary sinus and the right lower turbinate. The inferior part of the right middle turbinate was also excised. The neoplasm was separated from the right internal pterygoid muscle and the structures of the right pterygopalatine fossa (Figure 2). A part of the inferior wall of the right sphenoid sinus, the right half of the posterior nasopharyngeal wall, the superior half of the right eustachian tube, and the posterior one‐third of the nasal septum were removed en bloc. A Doppler ultrasound confirmed the location of the internal carotid artery (ICA) at the bottom of the neo‐cavity. Nasopharyngeal structures contralateral to the lesion were not removed.

## 4 CONCLUSION AND RESULTS

The postoperative course was uneventful and the patient was discharged the next day. Three weeks later, a new MRI confirmed the complete excision of the neoplasm. One year after surgical excision the patient remains free of local recurrence. However, he has distant metastasis to the liver and he is undergoing palliative chemotherapy.

## 5 DISCUSSION

Resection of recurrent carcinomas of the nasopharynx has been performed through open, robotic, and endoscopic approaches.^5^, ^6^, ^7^, ^8^ Minimally invasive approaches employed for managing recurrent carcinomas of the nasopharynx seem to have fewer cosmetic and functional morbidities.^5^, ^6^, ^7^, ^8^ Endoscopic nasopharyngectomy exhibits similar survival and local control outcomes to open approaches with less morbidity.^5^, ^10^ Vlantis et al.^8^ reported 100% 2‐year overall survival (OS) and 90% disease‐specific survival (DSS) for recurrent carcinomas of the nasopharynx treated with endoscopic nasopharyngectomy. Evidence from other studies suggests that an endoscopic nasopharyngectomy for early T‐classification recurrent carcinomas has similar oncologic outcomes to open approaches.^5^, ^11^ Adjacency to the ICA is not always a contradiction for surgery. Achieving negative resection margins and protecting ICA, renders the endoscopic surgical procedure challenging.^9^

Castelnuovo et al.^12^, ^13^ proposed a three‐type classification of endoscopic endonasal nasopharyngectomies. Tumor spreading determines the selection of the most appropriate approach. Type I is used for lesions limited to the posterior and superior wall of the nasopharynx, type II for lesions extended upward involving the anterior and inferior walls of the sphenoid sinus, and type III for lesions extended to the lateral wall and the parapharyngeal space.

Respecting the oncologic soundness rules is of paramount importance in head and neck cancer. Every tumor should be evaluated for its resectability bearing in mind that negative surgical margins improve the prognosis. Our case indicated that even in cases adjacent to critical structures, surgeons should explore the feasibility of surgery meticulously before declaring the neoplasm unresectable. Endoscopic nasopharyngectomy seems to be a safe and feasible procedure for managing recurrent nasopharyngeal malignancies with similar survival and local control outcomes to open approaches.

## AUTHOR CONTRIBUTIONS

**Paraskevi Karamitsou:** Conceptualization; data curation; formal analysis; investigation; methodology; resources; validation; visualization; writing – original draft. **Alexandros Poutoglidis:** Conceptualization; data curation; formal analysis; investigation; methodology; writing – review and editing. **Aikaterini Karamitsou:** Conceptualization; data curation; formal analysis; investigation; methodology; writing – review and editing. **Georgia‐Evangelia Papargyriou:** Formal analysis; investigation; methodology; supervision; validation; visualization; writing – review and editing. **Argyro Leventi:** Formal analysis; investigation; methodology; supervision; validation; visualization; writing – review and editing. **Christos Georgalas:** Investigation; methodology; resources; supervision; validation; visualization; writing – original draft.

## FUNDING INFORMATION

The authors received no financial support for this article's research, authorship, and/or publication.

## CONFLICT OF INTEREST STATEMENT

The authors declared no potential conflicts of interest concerning the research, authorship, and/or publication of this article.

## CONSENT

Written informed consent was obtained from the patient to publish this report in accordance with the journal's patient consent policy.

## Data Availability

Data sharing not applicable – no new data generated/Data sharing is not applicable to this article as no new data were created or analyzed in this study.
